# High dispersal capacity of *Culicoides obsoletus* (Diptera: Ceratopogonidae), vector of bluetongue and Schmallenberg viruses, revealed by landscape genetic analyses

**DOI:** 10.1186/s13071-020-04522-3

**Published:** 2021-02-03

**Authors:** Antoine Mignotte, Claire Garros, Simon Dellicour, Maude Jacquot, Marius Gilbert, Laetitia Gardès, Thomas Balenghien, Maxime Duhayon, Ignace Rakotoarivony, Maïa de Wavrechin, Karine Huber

**Affiliations:** 1 ASTRE, Univ Montpellier, Cirad, INRAE, Montpellier, France; 2 Cirad, UMR ASTRE, 34398 Montpellier, France; 3 Spatial Epidemiology Lab (SpELL), Université Libre de Bruxelles, CP160/12, 50, av. FD Roosevelt, 1050 Bruxelles, Belgium; 4 Department of Microbiology, Immunology and Transplantation, Rega Institute, KU Leuven, Herestraat 49, 3000 Leuven, Belgium; 5 UMR EPIA, Université Clermont Auvergne, INRAE, VetAgro Sup, 63122 Saint-Genès-Champanelle, France; 6 Cirad, UMR ASTRE, 97170 Petit-Bourg, Guadeloupe France; 7 Cirad, UMR ASTRE, 10100 Rabat, Morocco; 8 Unité Microbiologie, immunologie et maladies contagieuses, Institut Agronomique et Vétérinaire Hassan II, 10100 Rabat-Instituts, Morocco

**Keywords:** *Culicoides obsoletus*, Landscape genetics, Microsatellite, Dispersal, Palearctic region

## Abstract

**Background:**

In the last two decades, recurrent epizootics of bluetongue virus and Schmallenberg virus have been reported in the western Palearctic region. These viruses affect domestic cattle, sheep, goats and wild ruminants and are transmitted by native hematophagous midges of the genus *Culicoides* (Diptera: Ceratopogonidae). *Culicoides* dispersal is known to be stratified, i.e. due to a combination of dispersal processes occurring actively at short distances and passively or semi-actively at long distances, allowing individuals to jump hundreds of kilometers.

**Methods:**

Here, we aim to identify the environmental factors that promote or limit gene flow of *Culicoides obsoletus*, an abundant and widespread vector species in Europe, using an innovative framework integrating spatial, population genetics and statistical approaches. A total of 348 individuals were sampled in 46 sites in France and were genotyped using 13 newly designed microsatellite markers.

**Results:**

We found low genetic differentiation and a weak population structure for *C. obsoletus* across the country. Using three complementary inter-individual genetic distances, we did not detect any significant isolation by distance, but did detect significant anisotropic isolation by distance on a north-south axis. We employed a multiple regression on distance matrices approach to investigate the correlation between genetic and environmental distances. Among all the environmental factors that were tested, only cattle density seems to have an impact on *C. obsoletus* gene flow.

**Conclusions:**

The high dispersal capacity of *C. obsoletus* over land found in the present study calls for a re-evaluation of the impact of *Culicoides* on virus dispersal, and highlights the urgent need to better integrate molecular, spatial and statistical information to guide vector-borne disease control.
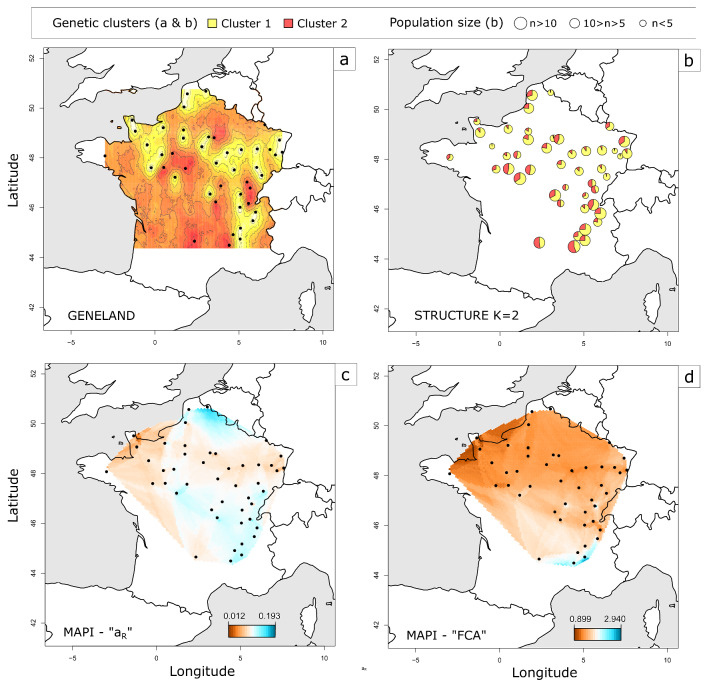

## Background

During a vector-borne disease outbreak, a precise understanding of the dispersal capacity of the vector species is key to implementing appropriate control strategies in order to limit the spread of the disease. Dipteran dispersal is known to be impacted by the composition and configuration of the landscape. When the dispersal of an organism is favored by a landscape characteristic (“environmental factor”) the latter can be categorized as a conductance factor [1]. On the contrary, when dispersal is limited by a landscape characteristic, the latter can be categorized as a resistance factor [1, 2]. However, some types of dispersal allow organisms to overcome resistance factors, as is the case for “stratified dispersion”. *Culicoides* dispersal is described as stratified, due to a combination of dispersal processes occurring actively at short distances, and passively or semi-actively at long distances [3].

Northern Europe experienced very sudden and rapid outbreaks of bluetongue virus (BTV) in 2006–2008 and Schmallenberg virus (SBV) in 2011–2012 [4]. Both viruses have spread very quickly and widely across the whole western Palearctic region transmitted by native *Culicoides* species. *Culicoides obsoletus* has been identified as the main vector species responsible for the transmission of BTV and SBV to wild and domestic ruminants in the western Palearctic region [5–7]. *Culicoides obsoletus* is a very widespread species, which is able to breed in a wide range of habitats [8, 9]. BTV spread is facilitated by favorable conditions for midge activity and viral replication, and the environmental and climatic drivers of BTV transmission in Europe have been suggested [10–14]. For example, high ambient temperatures reduce the incubation period of the virus [15], whereas high precipitation and wind speeds can reduce *Culicoides* flight activity [16–18].

Previous mark-release-recapture studies on *Culicoides* species showed that the post-release dispersal distance traveled during two nights ranges from 1 to 2.5 km, and is linked to the gradual search for hosts or oviposition sites [19]. The maximum recapture traveled distance recorded was 6 km for *Culicoides mohave* in a particular desert landscape [20]. Yet, the potentially high dispersal capacity of *Culicoides* and the high mortality of adults when they are manipulated represent practical limitations in the context of mark-release-recapture procedures. Many studies have reported a correlation between disease movement and wind-borne transport of *Culicoides* during outbreaks [3, 18, 21–26]. In 2017, Sanders et al. attempted to quantify *Culicoides* dispersal over land, and demonstrated through a capture-mark-recapture study that 84.4% of flights of more than 1 km took place downwind, while only 15.6% of flights were made upwind [27]. The introduction of BTV serotypes by wind-borne infected *Culicoides* has been demonstrated from northern Africa to southern Europe [28], from Kenya to Southwest Indian Ocean islands [29], from Sardinia to the Balearic Islands [25], from France to Corsica [30], in Ireland [31], and in the UK [32]. Most of these studies were supported by modeling analyses in which dispersal trajectories were evaluated by atmospheric dispersion models over water bodies [31, 33–37]. *Culicoides* dispersal over land has been investigated less than its dispersal over water [38], and little attempt has been made to link the former to environmental factors. Given the high dispersal capacity and the stratified dispersal pattern of *Culicoides*, it is crucial to determine inland connectivity among populations and to identify the potential environmental factors that promote or limit gene flows between them.

Classic approaches to studying the impact of landscape on gene flow generally use population-based sampling and are often based on a limited number of sampled populations [29, 39]. However, *Culicoides*, like other dipteran species, are not spatially structured into separate populations and must be considered as a continuum of individuals heterogeneously distributed across a landscape. In order to identify the environmental factors that have an impact on the dispersal of *Culicoides*, an individual approach is more relevant as it avoids the misinterpretation of inter-population genetic distance [40]. Indeed, the individual approach in landscape genetics aims at maximizing the number of sampling sites, and thus brings much greater statistical power to the detection of spatial patterns of genetic differentiation and the environmental factors that cause them [41]. In addition to the analysis of inter-individual genetic distances, population genetic structure can also be investigated, e.g. using gold standard Bayesian clustering methods. However, if isolation by distance occurs, these clustering analyses could overestimate the actual number of genetic clusters [42]. It is therefore important to also incorporate a visualization approach into the analytical workflow [43]. Mapping averaged pairwise information (MAPI) allows the visual comparison of genetic dissimilarity with some environmental factors, and also the development of working hypotheses. On the other hand, it is also necessary to use statistical analyses of genetic and environmental distances as a complement to MAPI.

The aims of the present study are to determine the inland connectivity of populations of a main vector species at large geographical scales and to identify the environmental factors that promote or limit gene flows between *Culicoides* populations in France. For this purpose, we characterized 13 microsatellite markers dedicated to *C. obsoletus*. We propose a complementary framework integrating multiple approaches which can be applied more generally to the study of gene flow and its links with environmental factors.

## Methods

### *Culicoides obsoletus* sampling and morphological identification

The data set analyzed in the present study is composed of 368 individual female biting midges collected in 46 sites in France in April 2011, using the national surveillance network for *Culicoides* populations or local complementary collections (Additional file 1: Table S1). Collections were made overnight using Onderstepoort Veterinary Institute light traps set up at farms near cattle or sheep. All insects were stored in 70% ethanol. Morphological identification to species level was carried out in the same way as described in a previous study [44] under a binocular microscope using available identification keys [45].

### DNA extraction, amplification, sequencing and sequence analyses

DNA was extracted from a total of 368 individuals belonging to the *C. obsoletus* species complex, and cytochrome* c* oxidase (COI) was amplified and sequenced following the procedure detailed in Mignotte et al. [44]. To ensure that only *C. obsoletus* individuals were included in the final data set [46], sequence assignation was run according to Mignotte et al. [44] (the reference COI sequences used for specific assignment of all individuals are available in Additional file 2: Table S2). All COI sequences were then aligned using the multiple sequence comparison by log-expectation algorithm [47] implemented in the software package GENEIOUS version 6.0.5 (Biomatters, http://www.geneious.com). In order to estimate unbiased gene flow of *C. obsoletus*, only individuals identified as such were used for further analyses.

### Development of microsatellite markers

Microsatellite markers were defined by next-generation sequencing following a similar protocol to the one described by Morillo et al. [48]. *Culicoides obsoletus* samples from France and the UK (Additional file 3: Table S3) were used as biological material to prepare a paired-end DNA library (Illumina Nextera technology) by using the Nextera DNA Sample Kit (ref. GAO9115; Illumina, San Diego, CA). The sequencing was performed on a MiSeq Sequencer [2 × 300-base pair (bp) read mode]. In order to maximize the length of the sequences, an assembly of the 2,299,075 reads was performed with the ABySS assembler [49]. The simple sequence repeat (SSR) loci search of di- and trinucleotide motifs was done with the MIcroSAtellite identification tool [50]. Primer design was done with the Primer3 software [51]. Among the 82,276 SSRs identified, primers were designed for 43,676 SSRs, of which 15,888 were dinucleotides, 23,656 were trinucleotides, and 4132 contained complex SSR motifs. For the SSR screening, we selected 3135 primer pairs flanking dinucleotide SSR motifs with a minimum of 12 repeats and amplifying fragments between 150 and 300 bp in length and a half-denaturation temperature close to 55 °C. We screened a set of 30 primer pairs and optimized both polymerase chain reaction (PCR) and multiplex PCR conditions.

### Microsatellite genotyping and loci filtering

Microsatellite makers were amplified by multiplex PCR with the Type-it-Microsatellites kit (Qiagen, Valencia, CA) according to the protocol described in the manufacturer’s manual and the annealing temperature given in Additional file 4: Table S4. Standard conditions for PCR amplification included an initial denaturation step at 95 °C for 5 min, 35 cycles of denaturation for 30 s at 95 °C, annealing for 1 min at variable temperature (Additional file 4: Table S4) and elongation for 1 min at 72 °C, followed by a final elongation of 5 min at 72 °C. Fragments were separated on an Applied Biosystems 3500xL Genetic Analyzer. Allelic size allocations for all individuals and microsatellite markers were performed using the program GeneMapper version 5 (Applied Biosystems, Life Technologies) with double blind reading to limit the potential interpretation bias from the reader.

A first validation of the polymorphisms of these 30 markers was carried out by amplifying and genotyping 48 individuals from various locations within the species geographic range. Then 13 loci showing good genetic profiles and clear allelic size variability were characterized as polymorphic markers and were selected for further analyses (Additional file 4: Table S4).

The previously extracted DNA of the 368 *C. obsoletus* was then amplified and genotyped at these 13 microsatellite loci. Genetic variability parameters, such as the number of alleles per locus, the allele size range, and the observed and expected heterozygosity were estimated per locus, and per population over the entire dataset using the R packages ggplot [52], poppr [53] and mmod [54]. Short allele dominance can be a source of heterozygote deficiency in a microsatellites data set [55, 56]. In order to avoid such bias, Spearman’s correlations rank between the size of the alleles and their frequencies were calculated in R [55]. Null allele frequencies were estimated using the R package PopGenReport [57], and a Bonferroni correction was applied to all matched tests to take into account potential biases associated with multiple comparisons [58]. Correlation between* F*_IS_ and null alleles was calculated in R, and loci with significant correlations were removed. The presence of an imbalance in genetic binding between each pair of loci was tested by Fisher’s exact test performed with the R package poppr [53]. The same R package was used along with the pegas R package to identify potential gaps in the panmictic matching regime. For this purpose, we performed chi-square tests to compare the observed heterozygosity deficits to the expected heterozygosity under the Hardy-Weinberg equilibrium with a significance threshold of 5%. We also calculated the fixation index (*F*_IS_) [59].

### Genetic clustering analyses

In order to determine the most likely number of genetic clusters (*K*) and the probability of assignment of each individual to these clusters, we used the Bayesian clustering methods implemented in the program STRUCTURE (version 2.3.4) [60] and GENELAND (version 4.5.0) [61]. The algorithm implemented in STRUCTURE infers genetic clusters that minimize deviations from Hardy-Weinberg equilibrium. In STRUCTURE, we specified the following settings: correlated allele frequency, an admixture model, and a locprior model. These options respectively allow one to (i) assume that allele frequencies are similar among populations; (ii) estimate for each individual the probability that it belongs to each* K* cluster; and (iii) use sampling locations as prior information to assist the clustering, which is particularly suitable when there is weak genetic structuring [62]. We performed ten independent runs for each value of* K* varying from 1 to 10. Each run consisted of a burn-in of 100,000 iterations in the Markov Monte Carlo chain (MCMC) and a stationary phase of 1,000,000 iterations in the MCMC. The most probable number of clusters was inferred with the ∆*K* method  (Evanno et al. 2005), *via* the STRUCTURE Harvester web platform [64]. This ∆*K* value is a way to determine the inflection in the [ln* P*(D)] curve. The clustering resulting from the Bayesian inference was transposed into percentages of assignment of each individual to the* K* inferred clusters and plotted on a map.

GENELAND also uses a Bayesian algorithm to infer population genetic clusters while taking into account the spatial position of individuals, making it a spatially explicit clustering method. The most probable number of clusters was also determined by running the algorithm with* K* ranging from 1 to 10. The analysis was based on 1,000,000 MCMC iterations with a thinning of 1000, maximum rate of the Poisson process fixed to 100, maximum number of nuclei in the Poisson-Voronoi tessellation fixed to 300 and a burn-in of 100. We used the R package graphics to produce a distribution map of genetic structure resulting from STRUCTURE and GENELAND analyses.

### Computation of genetic distances and mapping genetic dissimilarity

We computed three complementary inter-individual genetic distances. The first inter-individual genetic distance is based on a factorial correspondence analysis (FCA) performed with the program GENETIX version 4.05.2 [65]. The first ten FCA axes were used to calculate an Euclidean distance between all individuals using the R package ecodist [66], hereafter called the “FCA” distance [67]. The second inter-individual genetic distance is Rousset’s distance (*a*_R_), [68], which corresponds to* F*_ST_/(1 −* F*_ST_), but for pairs of individuals. We used the SPAGeDi 1.5 program [69] to compute* a*_R_ genetic distance. The third inter-individual genetic metric is Loiselle’s kinship coefficient (LKC) [70], which, unlike FCA and* a*_R_ that are measures of genetic dissimilarity, is an index of similarity [71]. We used the R package fields [72] to investigate isolation by distance by performing linear regressions and Mantel tests between inter-individual genetic distances and log-transformed geographic distances with R package fields [72]. Mantel tests [73] were performed with the R package vegan [74]. Inter-individual genetic distances were mapped using the program MAPI [43]. The method implemented in MAPI allows the visualization of spatial variation in genetic dissimilarity. It can also define areas where genetic dissimilarity is significantly lower or higher than expected by chance [43]. MAPI analyses were based on 1,000 permutations and an* α*-value of 0.05.

### Impact of environmental factors on genetic differentiation

We investigated the potential impact of several environmental factors on the genetic differentiation of *C. obsoletus*. In practice, we tested the association between matrices of pairwise genetic distances (see above) and matrices of environmental distances. These environmental distances were computed using the program Circuitscape 4.0.5 [75, 76] and were based on distinct environmental rasters (i.e. geo-referenced grids of environmental values) either treated as potential resistance (R) or conductance (C) factors:

i. Host densities: European index of distribution of roe deer (C) [77], European index of distribution of red deer (C) [77], global cattle, sheep and goat distributions in 2010 (C) [78].
ii. Terrestrial habitat: mean global elevation in 2010 (R) [79] and type of landscape cover (urban area, grassland, forest areas and croplands) (R). The four distinct land cover rasters (resolution: ∼ 1000 m) were generated from the Corine Land Cover 2012 raster (http://www.eea.europa.eu; resolution: ∼ 100 m) (Additional file 5: Fig. S1).
iii. Meteorological and climate data: mean surface temperature (R), mean precipitation (R), and mean wind speed and direction (C). We used two alternative rasters for each variable: April 2011 mean and 2000-2010 mean. Surface temperatures were obtained from monthly day and night land surface temperature means [80]. Precipitation and wind rasters were obtained from monthly means of precipitation, wind speed and the U and V components of wind (components of the horizontal wind towards the east and north) from the European Centre for Medium-range Weather Forecasts (Era5 Interim, http://www.ecmwf.int) over 10 years (2001–2010). Wind U and V components were used to compute prevailing meteorological wind direction (in degrees). Map and arrowheads indicating the mean wind direction over the 10 years at each raster pixel were generated with the R package rWind (Additional file 6: Fig. S2) [81].

A so-called null raster was also created and used as a negative control, i.e. a raster on which environmental distances computations correspond to a proxy of geographical distances. The null raster is a uniform raster with all cell values equal to 1. As in Dellicour et al. [84], three values of *k* (10, 100 and 1000) were used to modify the potential impact of the environment on the resistance/conductance value. We used multiple regression on distance matrices (MRDM) coupled with commonality analyses [82] to identify unique and common contributions of predictors to the variance in the environmental distance (response variable). All the analyses were carried out with the R packages ecodist [66] and yhat [83]. In addition, we also performed univariate analyses by comparing (i) the determination coefficient* R*^2^ obtained from the linear regression of genetic distances on distances computed on the environmental raster, and (ii) the determination coefficient* R*^2^ obtained from the linear regression of genetic distances on distances computed on the null raster. Only the environmental factors associated with a* R*^2^ higher than that obtained from the linear regression based on environmental distances computed on the null raster were selected for the multivariate analyses (MRDM-commonality analyses) [84]. The same criteria used to identify suppressors were used for the multivariate analysis as in the commonality analyses descriptions [80, 81]. These suppressors allow one to select environmental variables that are more explanatory than the geographical distance alone. An environmental variable is considered to be a suppressor if the regression coefficient and correlation, or the unique contribution and the common contribution, are of opposite signs [85].

### Anisotropic isolation by distance

Anisometric isolation by distance is a method used to identify directional gene flow and orientation at which the accumulation of genetic differentiation is the greatest. Thus the notion of anisotropy applied to isolation by distance means that the intensity and significance of isolation by distance varies according to the direction considered. In order to identify directional gene flows, we must calculate the projected distance between two sampling sites. The angle between sites was calculated using the R package geosphere [86] and transformed from degrees to radians. The set of angles between 0 and 360 were then tested as angles that maximize isolation by distance. The great-circle geographic distance between sampling sites was computed with the R package fields and then log transformed [72]. The projected distance matrix was calculated for each angle between all sampling sites, using the formula below. When calculating the projected geographical distance as a function of the angle between populations,* d*_AB_ is the geographical distance between population A and B and* a*_AB_ is the angle between populations A and B. 

1 $$log(|d_{AB}*cos(a_{AB})|)$$

A linear regression of the genetic distances on the projected geographical distances obtained for each angle was then performed. The angle that maximizes the* R*^2^ of this regression with a positive regression coefficient was considered as the angle maximizing the isolation by distance signal [87]. In order to confirm this analysis with a similar but complementary approach, we evaluated the direction that maximizes the correlation between geographic and genetic distance using the PASSaGE program and a bearing analysis. The workflow summarizing the genetic analyses is described in Additional file 7: Figure S3.

## Results

### Characterization of microsatellite markers and genetic diversity

A total of 368 COI sequences of *C. obsoletus* were obtained after the DNA barcoding step (Additional file 3: Table S3), and 368 *C. obsoletus* from 46 populations were genotyped with 13 microsatellite loci (Additional file 4: Table S4). A filter was also applied to exclude missing data. Individuals with more than 10% missing data were excluded from the analysis, i.e. 20 individuals. Loci OBSms25 and OBSms26 had the highest proportion of null alleles with 56% and 22%, respectively, but also the highest* F*_IS_ values of 0.691 and 0.357, respectively (Additional file 8: Table S5). The expected heterozygosity, which varied from 0.918 (OBSms5) to 0.404 (OBSms29), was always higher than the observed heterozygosity, which varied from 0.872 (OBSms13) to 0.197 (OBSms25), reflecting a heterozygous deficit (Additional file 8: Table S5). To ensure that all markers provided independent information, linkage disequilibrium between each pair of loci was tested. After Bonferroni correction, no significant linkage disequilibrium was observed between any loci. The OBSms25 locus was also the only locus with more than 5% missing data. Thus, only markers with a null percentage lower than 10% were kept for the final analysis. In view of the high proportion of null alleles and the strong correlation between* F*_IS_ and the rate of null alleles (*r* = 0.947, *p* > 0.001), the OBSms25 and OBSms26 loci were excluded from the analysis. No significant correlation between allele size and allele frequency was found for any of the 11 remaining loci (Additional file 8: Table S5). The genetic resolution of microsatellite markers depends on the number of markers and their polymorphisms. All 11 selected loci were therefore polymorphic (Additional file 8: Table S5), and the number of alleles present at a given locus varied from 40 for OBSms5 to nine for OBSms29. No significant isolation by distance was detected with the different inter-individual genetic distances,* a*_R_ (*R*^2^ < 0.001, *p* = 0.439; Additional file 9: Fig. S4), LKC (*R*^2^ < 0.001, *p* = 0.992; Additional file 9: Fig. S4), and FCA (*R*^2^ < 0.001, *p* = 0.361; Additional file 9: Fig. S4).

### Clustering analyses and mapping of genetic distances

**Fig. 1a–d:**
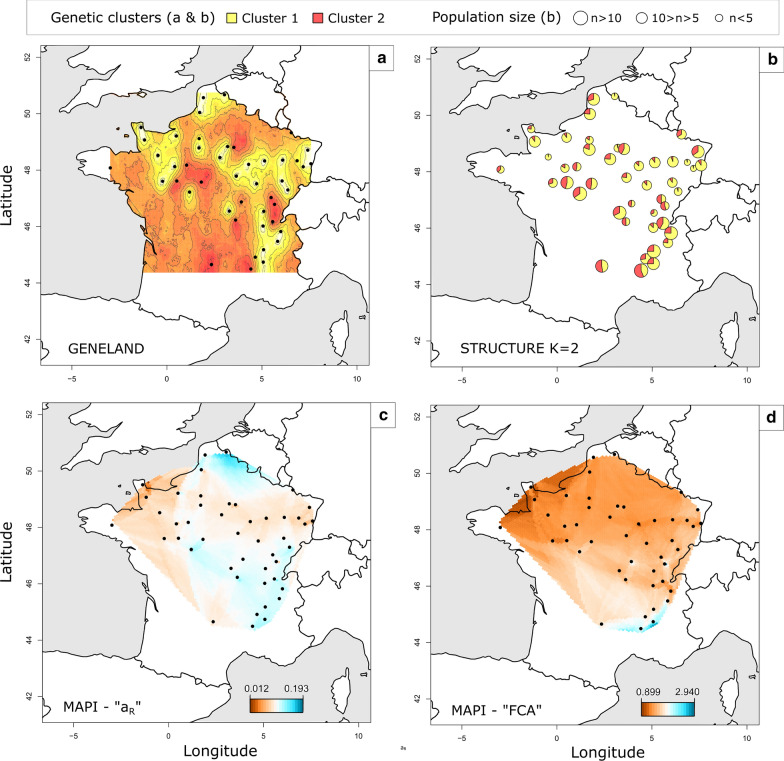
Genetic clustering and genetic differentiation of *Culicoides obsoletus*. Results of the genetic clustering analyses performed with GENELAND (**a**) and STRUCTURE (**b**), as well as smoothing of pairwise measures performed with mapping averaged pairwise information (*MAPI*) and based on (**c**) Rousset’s (*a*_R_) and (**d**) factorial correspondence analysis (*FCA*) inter-individual genetic distances. A specific color has been assigned to each genetic cluster in **a** and **b**. **c**, **d** Genetic dissimilarity is represented by a* color scale* ranging from* red* (lower genetic dissimilarity) to* blue* (higher genetic dissimilarity). The* black circles* indicate the sampling sites

The Bayesian analysis performed with STRUCTURE revealed a low genetic structuring of *C*. *obsoletus*. Meanwhile, the exact number of genetic clusters was not clearly defined, the optimal value of* K* was 2 (Additional file 10: Fig. S5), i.e. the co-existence of two genetic clusters was the most likely statistically (Fig. 1). The assignment of individuals to the different clusters revealed a weak genetic structuring of the populations. Although the assignment of a total of 284 out of the 348 *Culicoides* individuals to a genetic cluster was well supported (*Q* > 0.90), no clear association between spatial clustering and distribution was observed, i.e. there was no association between *Culicoides* sampling location and the genetic cluster distributions. However, a west-east transect seemed to emerge, as samples collected on this transect were more frequently assigned to cluster 1, contrary to the north-south transect which was more frequently represented by cluster 2. Genetic clusters inferred by GENELAND and STRUCTURE (*K =* 2) were consistent with these results, both clusters being found at all the sampling sites (Fig. 1a, b). Results for the second optimal* K* value (*K* = 4) of the STRUCTURE program gave a similar impression, reinforcing the conclusion that the populations are genetically weakly structured (Additional file 11: Fig. S6). Additional STRUCTURE analysis was carried out with only loci OBSms 4, 11 and 28, which are at Hardy Weinberg equilibrium, and no additional structure was detected using only these loci (data not shown). Genetic dissimilarity resulting from MAPI analyses with the* a*_R_ genetic distance showed a non-homogeneous pattern of dissimilarity. The maximum inter-individual genetic dissimilarity was 0.182 and the lowest was 0.002. The south of France was the most genetically dissimilar region. The center of France was also a zone of strong inter-individual dissimilarity, while eastern France showed low genetic dissimilarity. Another area spanning from the Paris region to the Alsace (northeastern France) showed low genetic dissimilarity (Fig. 1c, d). MAPI analyses with FCA inter-individual genetic distance showed a homogeneous genetic dissimilarity. However, almost none of the areas were significantly genetically dissimilar in MAPI analysis with the three genetic distances used.

### Investigating the impact of environmental factors

Univariate regressions revealed a significant positive association of FCA genetic distances with cattle distributions for *k* = 100 (*Q* = 0.009, *p* = 0.001) and *k* = 1000 (*Q* = 0.029, *p* = 0.001), with sheep distributions for *k* = 100 (*Q* = 0.002, *p* = 0.002) and for *k* = 1000 (*Q* = 0.006, *p* = 0.001), with elevation for *k* = 100 (*Q* = 0.001, *p* = 0.005) and for *k* = 1000 (*Q* = 0.001, *p* = 0.006), and with grassland for *k* = 100 (*Q* = 0.009, *p* = 0.001) and for *k* = 1000 (*Q* = 0.011, *p* = 0.001) distances (Additional file 12: Table S6). Similarly,* a*_R_ inter-individual genetic distances were significantly associated with distances computed from elevation for *k* = 100 (*Q* = 0.00018, *p* = 0.006) and for *k* = 1000 (*Q* = 0.00038, *p* = 0.007), with grassland for *k* = 100 (*Q* = 0.001, *p* = 0.001) and for *k* = 1000 (*Q* = 0.002, *p* = 0.001) and with urban areas for *k* = 100 (*Q* = 0.004, *p* = 0.001) and for *k* = 1000 (*Q* = 0.007, *p* = 0.001) rasters (Additional file 12: Table S6). However, no significant association was found between LKC genetic distance and the environmental distances (*Q* values always < 0).

Multivariate regressions revealed a significant positive association of FCA inter-individual genetic distance with cattle distributions for *k* = 1000 (*r* = 0.1733, *p* <0.001) and with grassland for *k* = 1000 (*r* = 0.1080, *p* <0.001) (Table 1; Additional file 12: Table S6). Also, multivariate regressions did not reveal any significant positive association of* a*_R_ inter-individual genetic distances with environmental distances. All environmental factors were considered as suppressors when using* a*_R_ to measure inter-individual genetic distances. Cattle distribution (*k* = 1000) with FCA inter-individual genetic distance was the only environmental factor that showed a unique contribution to the variance in the dependent variable higher than 1% (*U* = 0.02) (Additional file 12: Table S6).

**Table 1:** Results of multiple regression on distance matrices and additional parameters derived from commonality analysis

	*k*^a^	*r*	*β*	*U*	*C*
FCA					
Cattle density [C]^b^	1000	0.1733	0.1551	0.0221	0.0079
Grassland [R]^c^	1000	0.1080	0.0637	0.0037	0.0079
*a*_R_					
Elevation [R]^c^	100	0.0254	− 0.9945	0.00044	0.0002
Elevation [R]^c^	1000	0.0291	0.9860	0.00048	0.0004
Grassland [R]^c^	100	0.0448	0.2279	0.00005	0.0020
Grassland [R]^c^	1000	0.0539	− 0.1483	0.00002	0.0029
Urban areas [R]^c^	100	0.0725	− 0.1279	0.00014	0.0051
Urban areas [R]^c^	1000	0.0879	0.2156	0.00049	0.0072

Pearson’s correlation coefficient (*r*), significant regression coefficient (*β*), as well as unique (*U*) and common (*C*) contributions of environmental distances to the variance in the dependent variable are shown; *FCA* factorial correspondence analysis, *a*_R_ Rousset’s distance

^a^Re-scaling parameter used to transform the initial raster file

^b^The considered environmental raster was treated as a conductance [C] factor

^c^The considered environmental raster was treated as a resistance [R] factor

### Anisotropic isolation by distance

**Fig. 2a–c:**
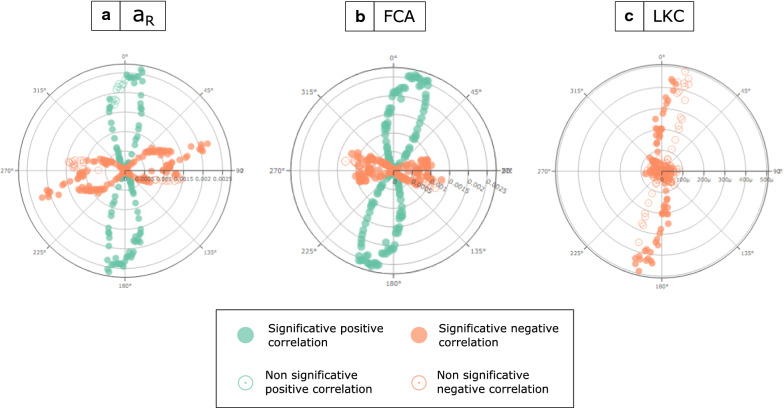
Results of anisotropic isolation by distance analyses. Polar plots show the correlation between geographical projected distances by angle and inter-individual genetic distances [*a*_R_ (**a**), Loiselle’s kinship coefficient (LKC) (**b**), and FCA (**c**)]. The set of angles between 0 and 360 were then tested as angles that maximize isolation by distance. The projected distance matrix was calculated for each angle between all sampling sites, using the formula in the Methods. A linear regression of the genetic distances on the projected geographical distances obtained for each angle was then performed. The angle that maximizes the* R*^2^ of this regression with a positive regression coefficient was considered as the angle maximizing the isolation by distance signal

**Fig. 3:**
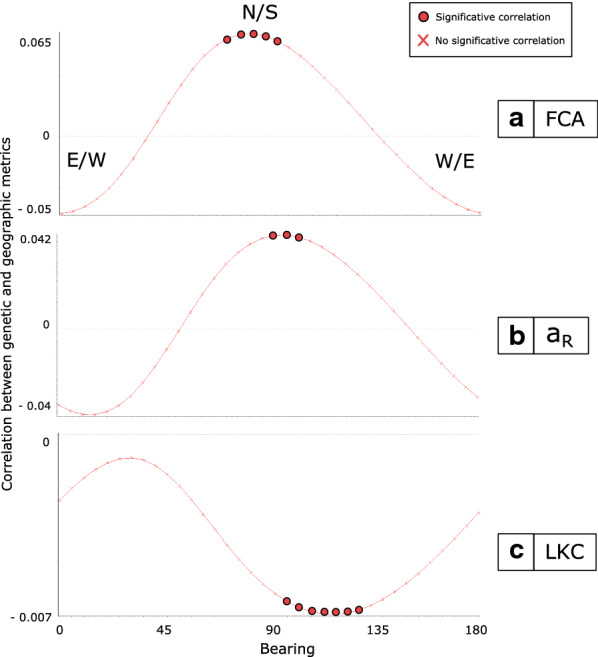
Bearing analysis: correlation between genetic [*a*_R_ (**a**), LKC (**b**), and FCA (**c**)] and geographical distances as a function of the angle between sampling sites.* Circles* indicate significant values,* crosses* indicate non-significant values*. E* East,* W* west,* S* south, N north; for other abbreviations, see Figs. 1 and 2

Globally, correlation between the genetic distance and the distance along the bearing *θ*, *d*_θ_, changed as a function of bearing *θ*. Anisotropic isolation by distance analyses revealed a significant positive correlation between* a*_R_ and FCA inter-individual genetic distances and orientational distances computed along the north–south orientation and a significant negative correlation between genetic distances and orientational distances computed along the west-east orientation (Fig. 2a, b). For the analysis with LKC, which is a variable of genetic similarity, north–south was the orientation that maximized the isolation by distance (Fig. 2c). Bearing analyses with PASSaGE software revealed a significant distance isolation on the north–south axis with the genetic distances* a*_R_ and FCA (Fig. 3a, b). A negative correlation between LKC genetic similarity and geographical distance was observed on the north–south axis (Fig. 3c).

## Discussion

To the best of our knowledge, this is the first extensive landscape genetic study carried out on a main vector species of interest for livestock diseases, *C. obsoletus*. Bayesian clustering analysis revealed a very weak genetic structure of *C. obsoletus* in France, and a low level of inter-individual genetic differentiation was observed. We assessed the impact of a wide range of environmental factors on this pattern. Univariate and multivariate analyses highlighted an absence, or a weak impact, of most of the tested environmental factors on inter-individual measures of pairwise genetic differentiation. No isolation by distance pattern was detected at the scale of France with the set of inter-individual genetic distances used. However, an anisotropic isolation by distance analysis revealed significant distance isolation on the north-south axis (Additional file 13: Figure S7).

### High level of gene flow between *C. obsoletus* populations

The low overall inter-individual genetic dissimilarity highlighted by our study reflected the high level of gene flow for *C. obsoletus*, and reinforces what has been described previously for other *Culicoides* species. In particular, high levels of gene flow have already been observed in France for *Culicoides imicola* [30] and in Australia for *Culicoides brevitarsis* [88]. Genetic studies on *C. imicola* in Europe revealed a high level of gene flow, as reflected by the inference of two large genetic clusters: a “central Mediterranean cluster” including Algeria, Sardinia, Corsica, and the Pyrénées-Orientales and Var departments of France, and a “western Mediterranean cluster” including Morocco, Spain, Portugal and Majorca [26]. In North America, similar results were also found for *Culicoides stellifer*. No barriers to gene flow could be identified in this species in the southeastern USA [89]. In addition, no isolation by distance has been observed at the scale of France. Identifying a genetic structure pattern is a great challenge when inter-individual genetic dissimilarity is low. Although STRUCTURE performs well at low levels of population differentiation (0.02 < *F*_ST_ < 0.10) by using prior population and correlated allele frequency models [90], when the differentiation is weaker, such as in the case of highly dispersed organisms, it may encounter difficulties [91]. This underlines the importance of using complementary tools of visualization approaches like MAPI. In addition, the use of an individual approach avoids artificially considering individuals grouped into populations solely due to the effect of the trapping sites [2, 92]. Although our method of analysis seems complementary and coherent for the detection of genetic structure, *C. obsoletus* is not genetically or geographically structured at the scale of France.

### Livestock densities as potential drivers of *C. obsoletus* gene flow

It is undeniable that high gene flow homogenizes the genetic diversity of *Culicoides*. This can be explained by active dispersal and host-seeking movements, as suggested by our results. While the unique contribution of the environmental factors tested was very small, the contribution of cattle density was the strongest that we detected. Host density as a conductance factor for *Culicoides* is obviously consistent with the biology of the species. These results are in line with those obtained in landscape genetics studies of BTV, which identified distributions of cattle and sheep as key factors in BTV dispersal [13]. In addition, previous studies showed that dairy cattle density was negatively correlated with BTV spread. Although paradoxical with respect to the previous conclusions, this could be explained by the fact that dairy cattle tend to cluster around the milking parlor and move little, and thus form a fixed feed source, which limits the spread of *Culicoides*. On the contrary, beef cattle disperse much more in pastures and thus could encourage the dispersal of *Culicoides* when they seek a source of blood [93]. In view of these results, and of the marked preference of certain species of *Culicoides* for cattle [94], and of BTV emergence or re-emergence events in cattle in the Netherlands in 2006 and in France in 2015 [95, 96], it seems crucial to survey and closely monitor the movements of *Culicoides* in the vicinity of beef cattle farms [94].

In addition, it has been shown that BTV spread is facilitated at low elevation, i.e. up to 300 m [39, 93]. Altitude, an environmental factor tested in this study, failed to explain the inter-individual genetic differentiation of *C. obsoletus*. Altitude, therefore, does not act as a barrier between sampling sites located at relatively low altitudes. It would thus be interesting to integrate into future studies sampling sites at high altitude, i.e. above 1000 m, which are feasible habitats for *C. obsoletus* as it has a large altitudinal range [97]. However, it is possible that altitude does not directly impact the dispersal activity of *Culicoides*, but only the replication or viral infection of BTV due to low temperature. *Culicoides obsoletus* is an extreme generalist and can take a blood meal from a wide range of hosts [98]. The study of phylogenetically close but ecologically very different species of *Culicoides*, such as *Culicoides chiopterus* [99], might then allow the identification of very different dispersal patterns and the importance of bovine density, *C. chiopterus* being exclusively dependent on cattle for egg-laying [100].

### Long-range dispersal of *C. obsoletus*

The most genetically dissimilar individuals were mainly from the southernmost populations of the sampling area. Multiple non-exclusive lines of argument might explain the significant anisotropic isolation by distance observed on a north–south axis in France.

First, the significant anisotropic isolation by distance could be explained by wind dispersal. Dispersion phenomena caused by wind currents, mainly over seas, have already been established for *Culicoides* [3, 18, 21–25, 30]. The resultant dispersal events can be described as passive and active, respectively, because recapture was achieved downwind and upwind of the prevailing wind direction [19], although the activity of *Culicoides*, and thus its active dispersion, is reduced when the wind speed is higher than 3 m/s [16]. Examination of the map of the average wind directions in France over the last 10 years provides potential explanations for this. It can be seen that the southernmost sampling sites (with the most dissimilar individuals) are located in an area where the wind direction is different from that in the rest of France. It should be noted that the diffusion of BTV, and thus the dispersion of *Culicoides*, has already been associated with wind direction. For example, 2% of BTV infections occurred at distances greater than 31 km [101, 102] during the 2006 epizootic. The south of France was initially sampled; however, poor conservation of *Culicoides* DNA did not allow us to perform barcoding and microsatellite genotyping of these populations. A study of this geographical area could thus complete the findings of the present study and potentially identify a stronger genetic structure. This would make it possible to decide whether the premise of differentiation observed in the present study is due to an older phylogeographic structure, different wind dispersion in this geographical area, or a random pattern. It is also questionable whether the indications of very weak genetic structure could be due to differences in genetic diversity between the southern and the northern populations. Lower genetic diversity in the north could then be a sign of an expansion process that contradicts the interpretation of large-scale dispersion [103, 104]. Indeed, the spatial expansion of a population is generally accompanied by gradients of reduction in the genetic diversity of the population during the expansion process, caused by serial founder effects, which create a genetic bottleneck through a founding event [105, 106]. Population sampling at the same scale could allow comparison and estimation of genetic diversity.

Second, anisotropic isolation by distance may be due to an artifact of the sampling methods used. Indeed, if the extent of the sampling varies depending on direction, the distances projected from the angles may represent different distance distributions and lead to the over-representation of the values of strong genetic metrics in the direction of the scatter plot and the hit of positive correlation signals. Moreover, the absence of correlation does not necessarily mean more gene flow but an absence of isolation by distance, which can also result from a strong drift and thus less gene flow; a drift which depends on both dispersion and population sizes. It is therefore essential to use as a complement—as we have done here and with which we have achieved a similar result—an approach that weights the geographical distances between populations according to their orientation with respect to a given angle axis passing through the barycenter, as implemented in PASSaGE.

### Considerations for future work

Our results underline the importance of the methodological development of wind dispersal models for *Culicoides* not only over water but also on land. However, studies of landscape genetics remain indispensable and complementary in order to improve the accuracy of predictive models for *Culicoides* dispersal over land through the integration of meteorological, landscape and activity-based parameters previously tested and validated in landscape genetics workflows. In addition, population sampling of the same species at the European level, i.e. sampling in a very large proportion of the known range of the species, is necessary to observe more marked structuring at the European level and to estimate more precisely the gene flow. For example, *C. imicola* in Europe is structured into two large genetic clusters, the European central cluster and the European western cluster [26]. Moreover, the phylogeographic history of *C. obsoletus* in view of its geographical distribution throughout Europe and North America has only been little explored. On the contrary, the study of the phylogeography of *C. imicola*, the main Afrotropical vector, has shown that its range is from the northern part of sub-Saharan Africa to the Mediterranean Basin [39]. This type of study would make it possible to estimate the effective size of the populations, a key factor in the dispersal of *C. obsoletus*. In future studies, the use of high-throughput sequencing approaches using markers such as double-digest restriction-site associated DNA sequencing can provide greater resolution in view of the large number of single nucleotide polymorphisms (SNPs) revealed at a local scale, and improve our understanding of the active and passive dispersal of *Culicoides*. It could also be relevant to include more microsatellite markers or SNPs to improve genetic resolution and observe the matching and assignment of each individual.

## Conclusions

To the best of our knowledge, this study provides the first complete landscape genetic analysis of *C. obsoletus*, a major vector species of animal viruses in Europe. This study shows that the genetic structure of populations at the scale of a country can become homogeneous through large-scale dispersion. Our results demonstrate, for *C. obsoletus*, a very high inland dispersal and vectorization capacity, which has to be taken into consideration in further work on vector competence and epidemiological modeling of disease transmission. Wind direction could be a key factor in the dispersal of many insect vector species. Futures studies should increase their geographical extent to cover the entire area of species distribution and enable a better understanding of the limits of *Culicoides* gene flow. In addition to the biological information presented here, this study highlights several important areas for the improvement of methodologies that may currently limit the inclusion of wind direction in landscape genetic analyses.

## Supplementary information

**Additional file 1: Table S1.** Sampling sites and associated genetic diversity.* n* Sample size,* Ho* observed heterozygosity,* Hs* expected heterozygosity,* FIS* fixation index.

**Additional file 2: Table S2.** Reference sequences used for specific assignation.

**Additional file 3: Table S3.** Origin and numbers of individuals used to build up the DNA library necessary for the development of the microsatellite markers.

**Additional file 4: Table S4.** Primers of the 13 microsatellite markers used to genotype *C. obsoletus* populations.* DYE* Fluorochrome,* TFm* half denaturation temperature in degrees,* bp* base pairs.

**Additional file 5: Figure S1.** Environmental variables tested as potential factors that could impact inter-individual genetic differentiation of *C. obsoletus* in France (raster cell resolution: 0.04 arcmin).

**Additional file 6: Figure S2.** Map of wind direction averaged from 2000 to 2010. Sampling sites are represented by* black points*. The* color scale* represents the wind direction from 0 to 360 ° from north. The* arrowheads* indicate the exact wind direction of each raster pixel.

**Additional file 7: Figure S3.** Analytical workflow.

**Additional file 8: Table S5.** Genetic diversity by locus.* Ho* Observed heterozygosity,* Hs* expected heterozygosity,* FIS* fixation index,* SAD* short alleles dominance.

**Additional file 9: Figure S4.** Isolation by distance analyses: density plots, Mantel tests and linear regressions performed with each inter-individual genetic distance considered in this study.

**Additional file 10: Figure S5.** Identification of the optimal number of genetic clusters (*K*) inferred by STRUCTURE using the δ(*K*) and* L*’(*K*) methods.

**Additional file 11: Figure S6.** Population genetic structure results by clustering analyses performed STRUCTURE. A specific color has been assigned to each inferred genetic cluster.

**Additional file 12: Table S6.** Results of univariate analyses: determination coefficients (*R*^2^) estimated from univariate regressions between genetic and environmental distances. [C] indicates that the considered environmental raster was treated as a conductance factor for the computation of environmental distances with circuit theory. [R] indicates that the considered environmental raster was treated as a resistance factor for the computation of environmental distances with circuit theory.* Q* Difference between environmental multiple regression on distance matrices (MRDM)* R*^2^ and null raster MRDM* R*^2^. The results for* K* = 10 are not shown as they were non-significant.

**Additional file 13: Figure S7.** Bar plots of population genetic structure results by clustering analyses performed by STRUCTURE for* K* = 2 and* K* = 4. A specific color has been assigned to each inferred genetic cluster.

## Data Availability

All data generated or analyzed during this study are included in this article and its additional files. The newly generated sequences have been submitted to the GenBank database under accession numbers MT828832-MT828844. All the cytochrome* c* oxidase subunit 1 sequences are available on request.

## Abbreviations

BTV: Bluetongue virus
SBV: Schmallenberg virus
MAPI: Mapping averaged pairwise information
COI: Cytochrome* c* oxidase
bp: Base pair
SSR: Simple sequence repeat
PCR: Polymerase chain reaction
FCA: Factorial correspondence analysis
*a*_R_: Rousset’s distance
LKC: Loiselle’s kinship coefficient
MRDM: Multiple regression on distance matrices
SNP: Single nucleotide polymorphism
